# Dermatofibroma: clinicopathological analysis of 239 cases^☆^

**DOI:** 10.1016/j.abd.2024.03.012

**Published:** 2024-11-08

**Authors:** Dilara İlhan Erdil, Cem Leblebici, Duygu Erdil, Vildan Manav, Vefa Aslı Erdemir, Ayşe Esra Koku Aksu

**Affiliations:** a Department of Dermatology, University of Health Sciences, İstanbul Training and Research Hospital, İstanbul, Turkey; b Department of Pathology, University of Health Sciences, İstanbul Training and Research Hospital, İstanbul, Turkey; c Department of Dermatology, Istanbul Medeniyet University, İstanbul, Turkey

Dear Editor,

Dermatofibroma is one of the most commonly encountered benign, cutaneous soft tissue tumors, usually located on the extremities. Lesions are often in the form of solitary, raised, brown, firm, cutaneous nodules. Histopathologically, dermatofibroma is characterized by the proliferation of uniform spindle-shaped fibroblasts and histiocytoid cells in the dermis, with spindle cells forming a storiform appearance.^1^, ^2^ There are sub-variants of dermatofibroma which are less frequently observed. These are lipidized, aneurysmal, hemosiderotic, cellular, clear cell, atrophic, epithelioid, and sclerotic subtypes.^2^, ^3^ Rarely, it can also present in only subcutaneous form without causing changes on the skin surface.^4^

Common dermatofibroma is frequently observed, and rare variants are seen in 20% of histopathologically evaluated dermatofibromas.^2^ Subtypes other than common dermatofibroma might have different clinical manifestations and may cause confusion in the differential diagnosis. Therefore, in our study, clinicopathological evaluation of cases diagnosed with dermatofibroma was aimed. Further evaluation of infrequent variants, their differential diagnosis, and correct clinical diagnosis of dermatologists about the rare variants were analyzed.

The medical records of 239 dermatofibroma cases from 232 patients with clinical and histopathological diagnoses in our dermatology clinic between the years of 2012‒2021 were evaluated retrospectively. Patients with suspicious clinical or histopathological diagnoses or who had no clinical photography were excluded from the study. The following characteristics were recorded:

Demographic characteristics: Age, sex.

Clinical characteristics: Type of dermatofibroma, location of the skin lesions, the color of the lesions.

Histopathological characteristics: subtype of the dermatofibroma.

Statistical method: Mean, standard deviation, median minimum, maximum, frequency, and ratio values were used in the descriptive statistics of the data. The distribution of variables was measured with the Kolmogorov-Smirnov test. Mann-Whitney *U* test was used in the analysis of independent quantitative data. The Chi-Square test was used to analyze independent qualitative data, and Fisher's exact test was used when the Chi-Square test conditions were not met. SPSS 28.0 program was used in the analysis.

Results of 239 dermatofibroma cases from 232 patients were included in our study. Patient demographic, clinical, and histopathological characteristics are shown in Table 1. 61.2% of the patients were female, and 38.7% were male, with a mean age of 40.7 ± 12.9. The most common site of involvement was the lower extremity (50.8%), followed by upper extremity involvement with 35.2%. The lesions had a mean size of 7 mm ± 6.3. Histopathologically, 84.9% of the cases were common subtypes, followed by 7.5% atrophic, 4.6% cellular, 1.6% lipidized, 0.8% aneurysmal and 0.4% sclerotic subtypes (Fig. 1, Fig. 2, Fig. 3).

**Table 1:** Clinical and histopathological characteristics of Dermatofibroma (DF) patients.

		Min‒Max	Median	Avg. ± Std dev	
Age		12.0**‒**72.0	40.0	40.77 ± 12.92	
Size (mm)		2.0 - 45.0	5.0	7.05 ± 6.31	

				N	%
Gender	Female			142	61.2
	Male			90	38.7
Location	Lower extremity			121	50.8
	Upper extremity			84	35.2
	Trunk			29	12.1
	Face			4	1.6
Clinical differential diagnosis of DF	(−)			30	12.5
	(+)			209	87.4
Classical DF Histopathology	(−)			36	15
	(+)			203	84.9
Histopathological sub-variant	Classical			203	84.9
	Atrophic			18	7.5
	Cellular			11	4.6
	Aneurysmal			2	0.8
	Lipidized			4	1.6
	Sclerotic			1	0.4

**Fig. 1:**
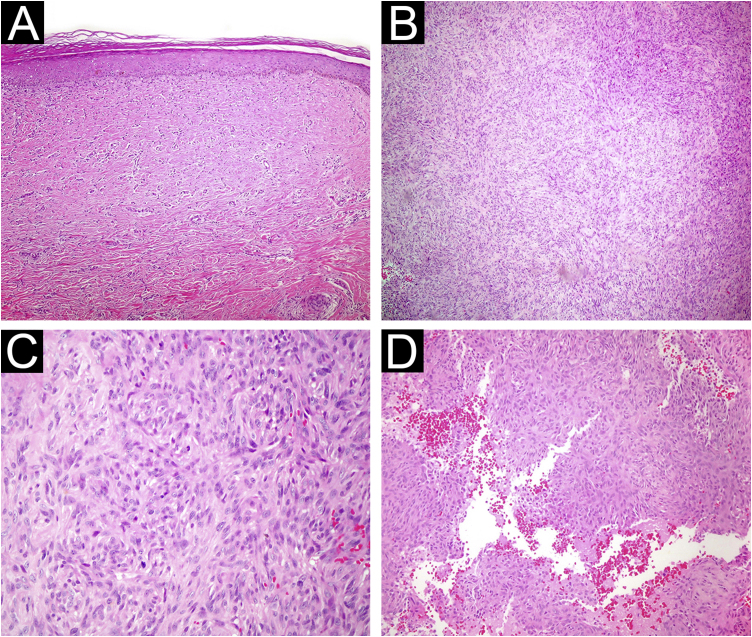
(A) Atrophic dermatofibroma. The lesion is hypocellular, and exhibits marked hyalinization. (Hematoxylin & eosin, ×200) (B) Cellular dermatofibroma with noticeable cellularity. (Hematoxylin & eosin, ×100) (C) High magnification of cellular dermatofibroma showing prominent mitotic figures. (Hematoxylin & eosin, ×400) (D) Aneurysmal fibrous histiocytoma. Cystic spaces filled with blood are observed within the lesion. (Hematoxylin & eosin, ×200).

**Fig. 2:**
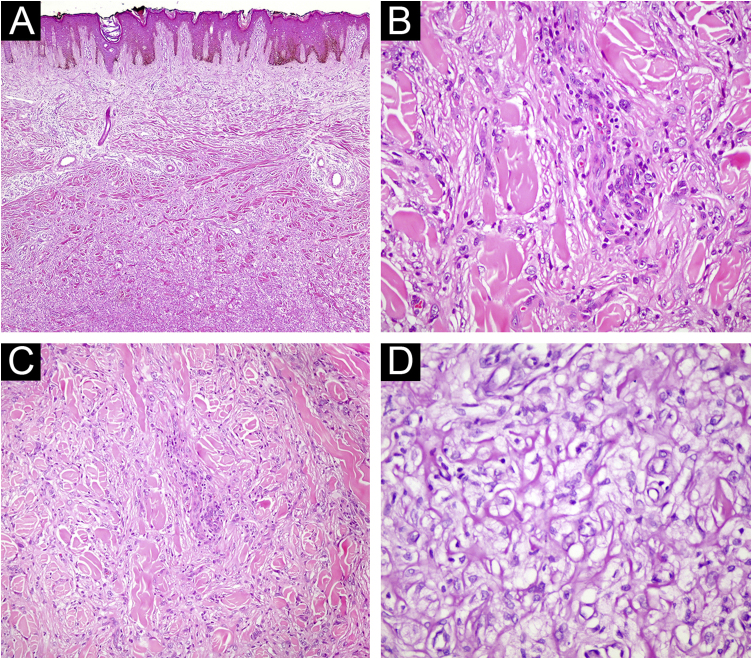
(A) Fibrous histiocytoma with atypical features. Marked nuclear pleomorphism is evident (Hematoxylin & eosin, ×200). (B) Fibrous histiocytoma with atypical features. Bizarre nucleated cells are observed (Hematoxylin & eosin, ×400). (C) Lipidized fibrous histiocytoma. At low magnification, significant hyalinization is noticeable in the lesion (Hematoxylin & eosin, ×40) (D) Foamy cytoplasmic appearance in the cytoplasm of lesional cells (Hematoxylin & eosin, ×400).

**Fig. 3:**
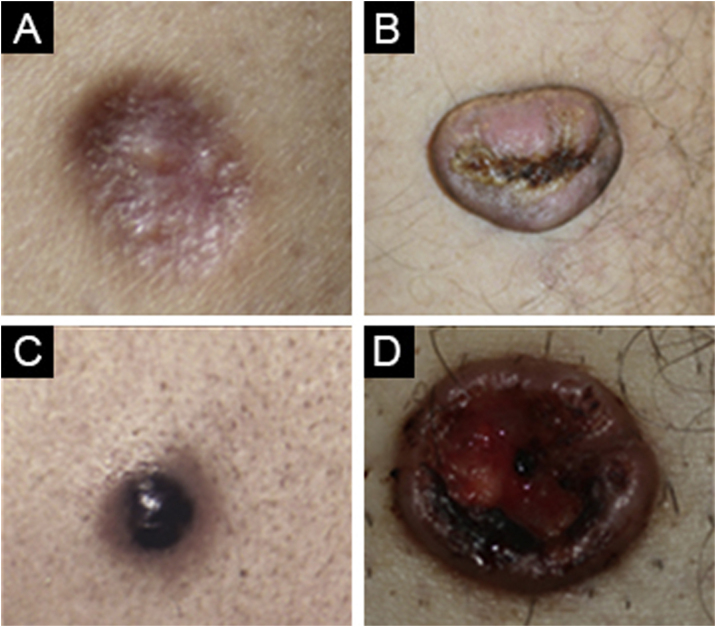
(A) Atrophic dermatofibroma. (B) Cellular dermatofibroma. (C) Aneurysmal dermatofibroma. (D) Lipidized dermatofibroma.

While dermatofibroma was considered the first diagnosis in 87.4% of the cases, dermatofibroma was not among the preliminary diagnoses in 12.5%. The most commonly considered differential diagnoses were foreign body granuloma, prurigo nodularis, pilomatricoma, keloid, and dermatofibrosarcoma protuberans with decreasing order (Fig. 4).

**Fig. 4:**
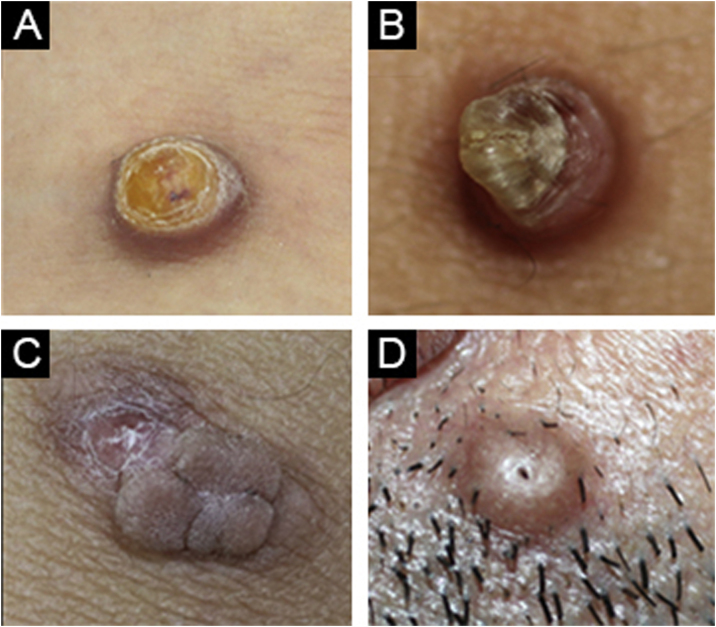
(A) Cellular dermatofibroma case with pre-diagnosis of eccrine poroma. (B) Common dermatofibroma case with a pre-diagnosis of keratoacanthoma. (C) Cellular dermatofibroma case with a pre-diagnosis of irritated skin-tag. (D) Common dermatofibroma case with a pre-diagnosis of trichofolliculoma.

The age, gender, and size of the lesion did not differ significantly (p > 0.05) between the groups with and without classical Histopathology (HP). The age of the patients in the group with differential diagnosis DF was significantly (p < 0.05) lower than the group without DF. Gender distribution did not differ significantly (p > 0.05) between groups with and without differential diagnosis of DF. The size of the lesion was significantly higher (p < 0.05) in the group with a pre-diagnosis of DF than in the group without a pre-diagnosis of DF. The rate of common histopathology in the group with differential diagnosis of DF was significantly higher (p < 0.05) than in the group without differential diagnosis of DF (Table 2).

**Table 2:** Dermatofibroma (DF) diagnosis according to clinical variables.

		Pre-diagnosis of DF(−) (n = 40)		Pre-diagnosis of DF(+) (n = 209)		
		Avg ± SD/n (%)	Median	Avg ± SD/n (%)	Median	p
Age		47.21 ± 13.73	50.0	39.85 ± 12.56	39.0	0.005^a^
Gender	Female	15 (38.5%)		127 (62.6%)		0.263^b^
	Male	14 (35.9%)		76 (37.4%)		
Size		5.17 ± 4.24	4.0	7.33 ± 6.51	5.0	0.043^a^
Classical DF	(−)	11 (36.7%)		25 (12.0%)		0.000^b^
	(+)	19 (63.3%)		184 (88.0%)		

a Mann–whitney *U* test.

b Chi-Square test.

Dermatofibroma is one of the most common benign skin tumors encountered in clinical practice. Although the diagnosis of dermatofibroma is straightforward, the diagnosis of rare histopathological subtypes may be challenging. In our study, rare variants were observed at a rate of 15%. It has been reported in the literature that dermatofibromas can mimic many different lesions, as they can be encountered in different colors and various clinical spectrums. Foreign body granuloma, prurigo nodularis, and pilomatricoma were the most common differential diagnoses in our clinical practice.

In a study that included 30 aneurysmal fibrous histiocytomas, intradermal nevi, leiomyomas, or benign vascular tumors were often considered at the initial diagnosis. Focal thrombosis and the amount of hemosiderin were thought to be the factors affecting the color of the presentation.^5^ Similarly, in our study, a case of aneurysmal fibrous histiocytoma presented with a black color and caused difficulties in the differential diagnosis.

Lipidized dermatofibroma may be in the form of a large exophytic yellow nodule, usually around the ankle, and may be clinically confused with juvenile xanthogranuloma. Lipidized dermatofibroma usually involves the lower extremities, while juvenile xanthogranuloma affects the head and neck region.^2^, ^6^ All of our lipidized dermatofibroma cases were localized in the lower extremities.

In terms of cellular dermatofibroma, the distinction from Dermatofibrosarcoma Protuberans (DFSP) gains importance. One of its distinctive features is that it can clinically involve different localisations, such as the face, hands and feet, apart from the extremities.^7^ The human progenitor cell antigen (CD34) positivity is up to 50%‒100% in DFSP, while focal positivity of CD34 can be detected at < 20% in DF.^8^ In a recent study, fat necrosis accompanied by lymphocytic infiltrate was seen in all cellular dermatofibroma cases, and its absence in DFSP cases shows that it can be helpful in histopathological diagnosis.^9^ While dermatofibroma was considered the initial diagnosis in 87.0% of the cases, dermatofibroma was not among the preliminary diagnoses in 12.5%. Among these cases, 17.0% of them were cellular dermatofibroma. Therefore differential diagnosis with DFSP or other malignancies is essential.

The main limitation of our study is its retrospective design. However, having photography of all our patients has made clinical assessment possible. The absence of other rare histopathological subtypes of dermatofibroma might be another limitation in our study.

In conclusion, dermatofibroma is one of the most common benign soft tissue tumors with a straightforward diagnosis. On the other hand, recognizing the rare variants of dermatofibroma might help clinicians to differentiate from the mimickers.

## Authors’ contributions

Dilara Ilhan Erdil: Approval of the final version of the manuscript; critical literature review, data collection, analysis and interpretation; effective participation in research orientation, intellectual participation in propaedeutic and/or therapeutic management of studied cases; manuscript critical review; preparation and writing of the manuscript; statistical analysis, study conception and planning.

Cem Leblebici: Approval of the final version of the manuscript; data collection, analysis and interpretation; effective participation in research orientation; ıntellectual participation in propaedeutic and/or therapeutic management of studied cases; manuscript critical review.

Duygu Erdil: Approval of the final version of the manuscript; data collection, analysis and interpretation; effective participation in research orientation; ıntellectual participation in propaedeutic and/or therapeutic management of studied cases; manuscript critical review.

Vildan Manav: Approval of the final version of the manuscript; data collection, analysis and interpretation; effective participation in research orientation; ıntellectual participation in propaedeutic and/or therapeutic management of studied cases; manuscript critical review.

Vefa Aslı Erdemir: Approval of the final version of the manuscript; data collection, analysis and interpretation; effective participation in research orientation; ıntellectual participation in propaedeutic and/or therapeutic management of studied cases; manuscript critical review.

Ayşe Esra Koku Aksu: Approval of the final version of the manuscript; critical literature review; data collection, analysis and interpretation; effective participation in research orientation; ıntellectual participation in propaedeutic and/or therapeutic management of studied cases; manuscript critical review; preparation and writing of the manuscript; statistical analysis; study conception and planning.

## Financial support

None declared.

## Conflicts of interest

None declared.

## References

[1] Han T.Y., Chang H.S., Lee J.H., Lee W.M., Son S.J. (2011). A clinical and histopathological study of 122 cases of dermatofibroma (benign fibrous histiocytoma). Ann Dermatol..

[2] Alves J.V., Matos D.M., Barreiros H.F., Bartolo E.A. (2014). Variants of dermatofibroma ‒ a histopathological study. An Bras Dermatol..

[3] Felty C.C., Linos K. (2019). Epithelioid fibrous histiocytoma: a concise review. Am J Dermatopathol..

[4] Lee W.J., Jung J.M., Won C.H., Chang S.E., Choi J.H., Moon K.C. (2015). Clinical and histological patterns of dermatofibroma without gross skin surface change: a comparative study with conventional dermatofibroma. Indian J Dermatol Venereol Leprol..

[5] Nabatanzi A., Male M., Qu X.Y., Li Y.Q., Meng X., Di W.S. (2019). Aneurysmal fibrous histiocytoma: clinicopathology analysis of 30 cases of a rare variant of cutaneous fibrohistiocytoma. Curr Med Sci..

[6] Seo J.K., Shin E.J., Jeong K.H., Shin M.K. (2019). Lipidized fibrous histiocytoma: differential diagnosis from juvenile xanthogranuloma. Ann Dermatol..

[7] Luzar B., Calonje E. (2010). Cutaneous fibrohistiocytic tumours - an update. Histopathology..

[8] Abenoza P., Lillemoe T. (1993). CD34 and factor XIIIa in the differential diagnosis of dermatofibroma and dermatofibrosarcoma protuberans. Am J Dermatopathol..

[9] Schechter S.A., Bresler S.C., Patel R.M. (2020). Fat necrosis with an associated lymphocytic infiltrate represents a histopathologic clue that distinguishes cellular dermatofibroma from dermatofibrosarcoma protuberans. J Cutan Pathol..

